# Celecoxib enhances the sensitivity of non-small-cell lung cancer cells to radiation-induced apoptosis through downregulation of the Akt/mTOR signaling pathway and COX-2 expression

**DOI:** 10.1371/journal.pone.0223760

**Published:** 2019-10-15

**Authors:** Pan Zhang, Dan He, Erqun Song, Mingdong Jiang, Yang Song

**Affiliations:** 1 Key Laboratory of Luminescence and Real-Time Analytical Chemistry, Ministry of Education, College of Pharmaceutical Sciences, Southwest University, Chongqing, P. R. China; 2 Department of Oncology, Nuclear Industry Hospital, Chengdu, Sichuan, P. R. China; 3 Department of Radiation Oncology, The Ninth People's Hospital of Chongqing, Chongqing, P. R. China; Duke University School of Medicine, UNITED STATES

## Abstract

The current study aimed to identify the radiosensitizing effect of celecoxib, a selective cyclooxygenase-2 (COX-2) inhibitor, in combination with radiotherapy in non-small-cell lung cancer (NSCLC) cells. The combination of celecoxib potentiated radiation-induced apoptosis; however, no changes in cell cycle distribution and number of phosphorylated histone H2AX foci were detected, indicating a DNA damage-independent mechanism. In an *in vivo* mouse model, the tumor size was significantly decreased in the group combining celecoxib with radiation compared with the radiation only group. Phosphorylation of protein kinase B (Akt) and mammalian target of rapamycin (mTOR), as well as expression of COX-2 were significantly downregulated in cells treated with the combination of celecoxib and radiation compared with the radiation only group. The result indicated that celecoxib exhibits radiosensitizing effects through COX-2 and Akt/mTOR-dependent mechanisms. Induction the Akt/mTOR signaling pathway promotes radioresistance in various cancers, including NSCLC. Therefore, the current study suggested the therapeutic potential of combination therapy of celecoxib and radiation in the prevention of radioresistance.

## Introduction

Lung cancer, particularly non-small-cell lung cancer (NSCLC), is one of the most common cancers worldwide [1]. Radiotherapy is a promising treatment strategy for enhancing the survival time of patients and promoting quality of life [2]. However, the development of radioresistance and severe side effects on normal tissues frequently result in failure to apply radiotherapy. Therefore, it is critical to develop an improved strategy to overcome radioresistance in lung cancer.

An ideal radiosensitizer is expected to have low or absent toxicity to normal cells. However, most radiosensitizers used in clinic, including nitroimidazoles, fluorouracil, cisplatin and Taxol, do not match this criterion due to high toxicity to normal tissues. Therefore, various approaches have been explored to develop potent radiosensitizers to establish more suitable treatment strategies. Growing evidence indicates that inflammation serves an important role in modulating radiation responsiveness of cells [3]. Inflammation suppresses the effectiveness of radiotherapy [4] and substantially contributes to the development and progression of cancer [5]. The goal of novel therapeutic approaches is to disrupt proinflammatory cytokines and to stimulate receptors and signaling cascades. The aim of the current study was to develop a new strategy to increase sensitivity to radiation.

Nonsteroidal anti-inflammatory drugs (NSAIDs) are used worldwide for the treatment of pain, inflammation and fever. Cyclooxygenase-2 (COX-2) is an important rate-limiting enzyme in prostaglandin synthesis and interferes with angiogenesis and metastasis [6–8]. Previous studies have reported that the inhibition of COX-2 is beneficial for chemotherapy [8, 9]. Notably, COX-2 overexpression is frequently observed in human premalignant, malignant and metastatic epithelial tumors, including lung, breast, colorectal and prostate cancer [10, 11]. Suppression of COX-2 has been proposed to be associated with chemopreventive effects of NSAIDs. However, whether NSAIDs serve a role in the resistance of radiotherapy is currently unknown.

Celecoxib belongs to the NSAIDs family and is a potent and COX-2-specific inhibitor. NSAIDs are commonly linked to side effects, including bleeding and perforation in the gastrointestinal system in chronic NSAID users [12]. Celecoxib has been demonstrated to be less toxic compared with traditional NSAIDs [13]. Preclinical studies have reveled that COX-2 inhibitors lower the proliferation of human lung cancer cells in combination with chemotherapy [14]; however, whether the combination of COX-2 inhibitors with radiotherapy has a better effect in NSCLC has not been investigated in clinical trials or laboratory studies. A previous study suggested that anticancer effects of celecoxib are independent of COX-2 inhibition [15]. Later mechanistic studies indicated that celecoxib exhibits proapoptotic effects by inhibiting 3-phosphoinositide-dependent kinase-1 (PDK-1) and the downstream protein kinase B (Akt) signaling pathway in human colon cancer cells [16]. A recent study demonstrated that celecoxib downregulates specificity protein 1 by inhibiting c-Jun N-terminal kinase, thus enhancing the radiation sensitivity and inhibiting the migration and invasion of cancer cells [17]. To confirm this assumption, the current study evaluated effects of celecoxib on radiation response of NSCLC cells. In addition, possible underlying cellular mechanisms were investigated. The current study suggested the therapeutic potential of combination therapy of celecoxib and radiation in the prevention of radioresistance.

## Materials and methods

### Chemicals and reagents

Celecoxib was purchased from Dalian Meilun Biology Technology Co., Ltd. (Dalian, China), dissolved in dimethyl sulfoxide (DMSO; 10 mM) and diluted with ddH_2_O immediately prior to each experiment. The final concentration of DMSO was <0.2%. Procedures of celecoxib preparation were previously described [18]. The Cell counting kit-8 (CCK-8) and the terminal transferase-mediated dUTP-biotin nick end labeling (TUNEL) assay kit were purchased from Beyotime Institute of Biotechnology (Nanjing, China). L-glutamine, penicillin and streptomycin were purchased from Aladdin Reagent Co., Ltd. (Shanghai, China). All antibodies details are reported in Table 1. All chemicals used were of the highest commercial grade.

**Table 1:** Antibody details.

Protein	Cat. no.	Dilution	Supplier
γ-H2AX	D155226	1:300	Sangon Biotech Co., Ltd.^a^
p-Akt	D151416	1:300	Sangon Biotech Co., Ltd.^a^
Akt	D120056	1:300	Sangon Biotech Co., Ltd.^a^
p-mTOR	D151417	1:300	Sangon Biotech Co., Ltd.^a^
mTOR	D160640	1:300	Sangon Biotech Co., Ltd.^a^
β-actin	D195301	1:300	Sangon Biotech Co., Ltd.^a^
Cyclin B1	55004-1-AP	1:500	ProteinTech Group, Inc.^b^
COX-2	12375-1-AP	1:500	ProteinTech Group, Inc.^b^
Parp1	13371-1-AP	1:500	ProteinTech Group, Inc.^b^
caspase 3	19677-1-AP	1:500	ProteinTech Group, Inc.^b^
Bcl-2	12789-1-AP	1:500	ProteinTech Group, Inc.^b^
Alexa Fluor555	A-21428	1:250	Invitrogen^c^
IgG-HRP	D110117	1:1,000	Sangon Biotech Co., Ltd.^a^

^a^Sangon Biotech Co., Ltd., Shanghai, China

^b^ProteinTech Group, Inc. Chicago, IL, USA

^c^Invitrogen; Thermo Fisher Scentific, Waltham, MA, USA.

γ-H2AX, phosphorylated histone H2AX; COX-2, cyclooxygenase-2; mTOR, mammalian target of rapamycin; Akt, protein kinase B; p-, phosphorylated; HRP, horseradish peroxidase.

### Cell culture

NSCLC cell lines A549, H1299, L78 and PGCL3 were obtained from The Third Military Medical University (Chongqing, China). Cells were maintained in Dulbecco’s modified Eagle’s medium (Gibco; Thermo Fisher Scientific, Inc., Waltham, MA, USA) supplemented with 10% fetal bovine serum (Hangzhou Sijiqing Biological Engineering Materials Co., Ltd., Hangzhou, China), L-glutamine (2 mM), penicillin (100 U/ml) and streptomycin (100 μg/ml) at 37°C with 5% CO_2_ in an humidified incubator. Cell numbers were determined using a hemocytometer. Irradiation was performed using a cobalt-60 gamma-ray irradiator at 2–6 Gy/min.

### Cell viability assay

A549, H1299, L78 and PGCL3 cells were seeded in 96-well culture plates at 1x10^4^ cells/well. Cells were pretreated with 2 or 5 μM of celecoxib for 24 h and then irradiated at 2, 4 and 6 Gy/min for 1 min; The maximum dosage of 6 Gy was selected based on a previous publication [19]. Celecoxib concentrations were adapted from a previous publication [18]. Following radiation, cells were cultured for 24 h, medium was removed and replaced with CCK-8 solution and cells were incubated for further 4 h. A total of 10 μl kit reagent was added to each well and samples were incubated for another 3 h. The optical density (OD) value determined at 450 nm using a microplate reader.

### Clonogenic survival assay

A549 cells (1x10^5^/well) were plated in collagen-coated 6-well plates and incubated overnight to adhere. Cells were pretreated with celecoxib (5 μM) for 24 h and then irradiated at 2 Gy/min for 1 min. Cells were cultured for 24 h and replaced with drug-free medium. The cells were then maintained at 37°C for 14 days to allow for the formation of colonies,fixed in 4% paraformaldehyde for 5 min at room temperature, washed three times with PBS and stained with pure methylene blue [2 g/l in 1:1 (v/v) EtOH/H_2_O] for 15 min. Colonies with >50 cells were selected and counted under a microscope. Synergism graphs on colony reduction were constructed by Microsoft Excel 2016.

### Cell cycle arrest assay

To distinguish cells in various cell cycle phases by flow cytometry, the DNA content was quantified using propidium iodide (PI). Following incubation of A549 cells (1x10^5^/well) with celecoxib (5 μM) for 24 h, cells were irradiated at 2 Gy/min for 1 min, cultured for 24 h, harvested and washed twice with PBS. Cells were fixed overnight in 70% ethanol at 4°C and stained with PI (5 μg/ml) for 30 min at 4°C in the dark. Fluorescence of PI was recorded (excitation, 488 nm; emission, 530 nm) by flow cytometer. DNA content and cell cycles were analyzed using a flow cytometer and the percentage of cells in each phase was calculated by FlowJo 10.0.

### Apoptosis assay

A549 cells (1x10^5^/well) were preincubated with celecoxib (5 μM) for 24 h, irradiated at 2 Gy/min for 1 min, cultured for 24 h, harvested and washed with ice-cold PBS. Then, cells were trypsinized and resuspended in binding buffer (10 mM HEPES, pH 7.4, 140 mM NaCl, 1mM MgCl_2,_ 5mM KCl, 2.5 mM CaCl_2_). Aliquots (100 μl) of cell solution were mixed with 5 μl Annexin V- fluorescein isothiocyanate (BD Biosciences, San Jose, CA, USA) and 10 μl PI (stock concentration, 50 μg/ml) and incubated in the dark for 2 h at room temperature. Binding buffer (400 μl) was added to each sample and cells were analyzed by a FACScan flow cytometer. The percentage of apoptotic cells was calculated with >10,000 cells/sample and data analysis was performed using CellQuest 5.1 software (BD Biosciences).

### Immunocytochemistry for phosphorylated histone H2AX (γ-H2AX) foci detection

Immunocytochemistry was performed to determine the nuclear distribution of γ-H2AX in radiated cells. Following preincubation of A549 cells (1x10^5^/well) with celecoxib (5 μM) for 24 h and irradiation at 2 Gy/min for 1 min, cells were cultured for further 24 h. Cells were then fixed with 4% paraformaldehyde for 5 min at room temperature and permeabilized with 0.2% Triton X-100 for 10 min at room temperature in PBS. Following blocking with 10% nonfat milk for 30 min at room temperature, cells were incubated with primary antibody against γ-H2AX (dilution, 1:300) at 4°C for 12 h. Then, cells were incubated with secondary antibody conjugated with Alexa Fluor555 for 1 h at 37°C. Cells were incubated with DAPI (5 μg/ml) for 15 min at room temperature to confirm the distribution of foci. The morphology was observed by fluorescence microscopy (40x).

### TUNEL assay

A549 cells (1x10^5^/well) were pretreated with celecoxib (5 μM) for 24 h and irradiated at 2 Gy/min for 1 min. Following culturing for 24 h, cells were fixed in ice-cold 4% paraformaldehyde for 5 min at room temperature, washed with PBS and analyzed for apoptotic cells using a TUNEL assay kit following the manufacturer’s instructions.

### Animal experiments

A total of 18 male BALB/c nude mice (age, 4–6 weeks; weight, 18–24 g) were used and the experimental protocols were approved by the Animal Care Committee of Southwest University (Chongqing, China) (#2017-M086) and were in accordance with the guidelines of National Institutes of Health guidelines. Several efforts were conducted to minimize animal suffering, including socially housed together (<6) indoors in suspended stainless steel cages in a temperature-regulated room (~22°C) with 12-h light/dark cycles, free access to water and standard mouse chow *ad libitum*. Tumor xenografts were established in nude mice that were injected with 1 x 10^7^ A549 cells in the flank and tumors were allowed to develop. When the tumor volume reached 75 mm^3^, mice were randomized into 3 groups (n = 6/group). Celecoxib (20 mg/kg body weight) was administered at the tumor site 1 h prior to irradiation; the dose was adjusted from a previous publication [20]. Mice in the irradiation and irradiation+celecoxib groups were irradiated with 10 Gy three times with a 7-days of interval. Tumor length and width were measured with a caliper every day and tumor volumes were calculated with the following formula: length (mm) x (width [mm])^2^ x0.52. The standard protocol entailed euthanasia of mice by CO_2_ inhalation, euthanasia was achieved by delivering 100% CO_2_ for a minimum of 3 min from a pressurized system into an enclosed non-precharged cage containing the animal, followed by exsanguination. Tumors were removed and homogenated for further study.

### Western blotting analysis

A549 cells were seeded in a dish (60 mm) at 1x10^6^ cells/ml using 5 ml full medium and were cultured for 24 h to adhere. Following the pretreatment with celecoxib (5 μM) for 24 h and irradiation at 2 Gy/min for 1 min, cells were cultured for further 24 h. Cells were harvested, washed with ice cold PBS and lysed with radioimmunoprecipitation assay buffer (50 mM Tris-HCl pH 7.4, 150 mM NaCl, 1 mM EDTA, 1% Triton X-100, 0.1% SDS, 1% sodium deoxycholate and 1 mM phenylmethylsulfonyl fluoride) for 30 min on ice. For animal study, tumor tissues were homogenated. Proteins (80 μg) were resolved on 8 or 10% SDS-PAGE gels and transferred to nitrocellulose membranes. The blots were blocked with 5% dried skim milk dissolved in TBST (10 mM TBS plus 1.0% Tween 20) for 1.5 h at room temperature. Membranes were incubated with primary antibodies for 3 h at 37°C followed by incubation with secondary goat anti-rabbit IgG-horseradish peroxidase-conjugated antibody for 2 h at 37°C (Table 1). Antibody binding was detected using enhanced chemiluminescence reagents.

### Statistical analysis

Data are presented as the mean ± standard deviation (n≥3). All data were analyzed using SPSS 19.0 (IBM Corp., Armonk, NY, USA). Statistical significance was determined using Student’s t-test or ANOVA. Tukey’s test was used in post hoc analysis for ANOVA. A probability value of p<0.05 or p<0.01 was considered to be statistically significant.

## Results

### Celecoxib improves radiosensitivity of NSCLC cells

**Fig 1:**
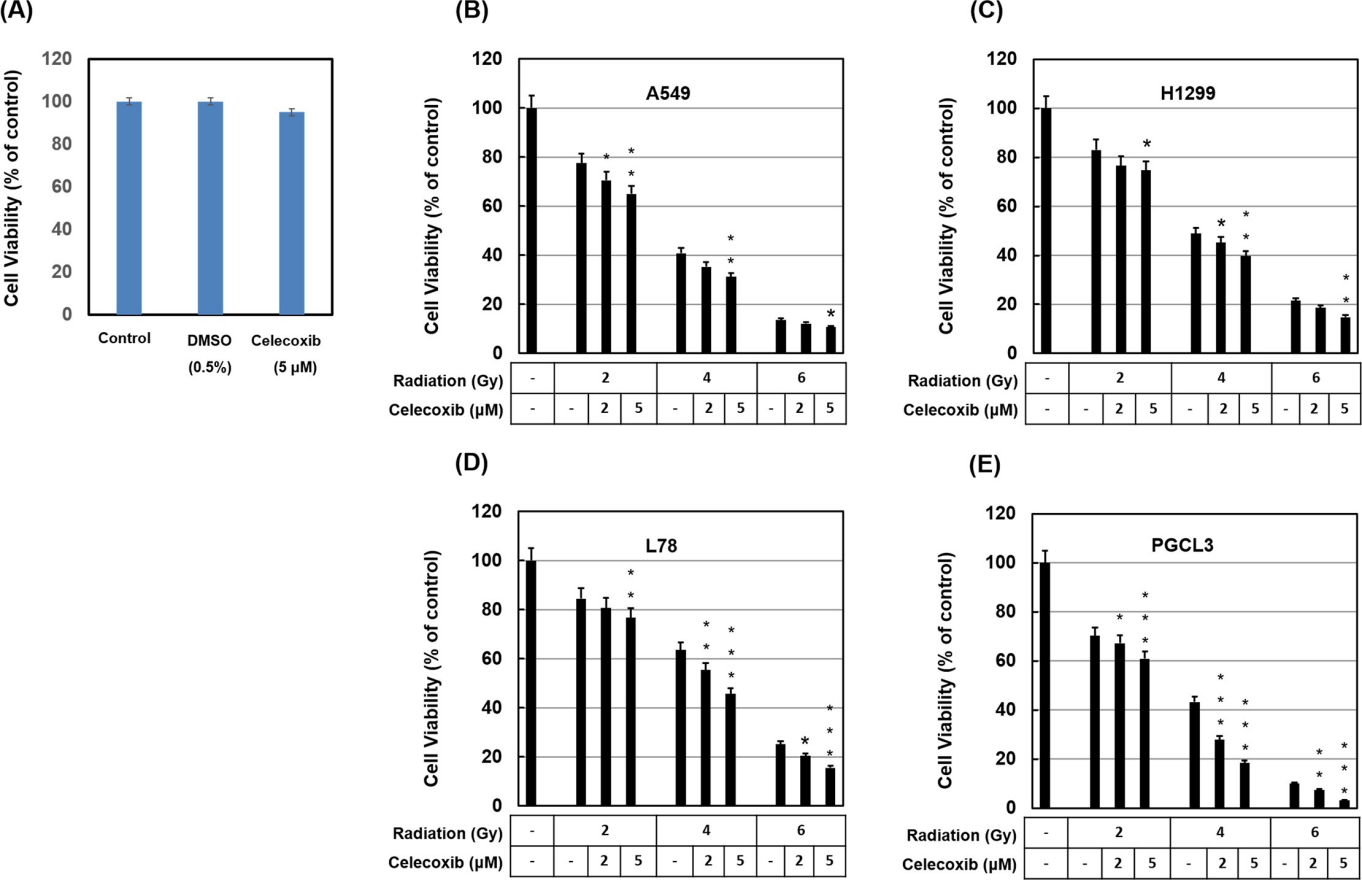
Effect of celecoxib and radiation on the cell viability was measured by CCK-8 assay. (A) Celecoxib shows no cytotoxicity at 5 μM concentration. (**B**) A549, (**C**) H1299, (**D**) L78 and (**E**) PGCL3 cells were treated with different concentrations of Celecoxib and then irradiated 24 h for indicated times. Data were shown as the means ± S.D. of three independent experiments. ***P<0.001, **P<0.01 and *P<0.05 indicated significant difference between radiation alone with radiation plus Celecoxib groups.

First, the cytotoxicity effect of celecoxib was determined in A549 cell lines. As shown in Fig 1A, 5 μM of celecoxib showed no significant cytotoxicity. To determine whether celecoxib improved radiosensitivity, various NSCLC cell lines, including A549, H1299, L78 and PGCL3, were treated with celecoxib and exposed to varying doses of radiation (2–6 Gy) for 24 h. According to an existing reference, patients with locoregionally advanced nasopharyngeal carcinoma received a total radiation dose of 72–76 Gy in 36–38 fractions to the primary lesion and 60 Gy in 30 fractions to cervical lymph-node lesions and celecoxib was administered at escalating doses of 400, 600 and 800 mg/day [21]. Therefore, the current study established lower doses compared with clinical practice.

**Fig 2:**
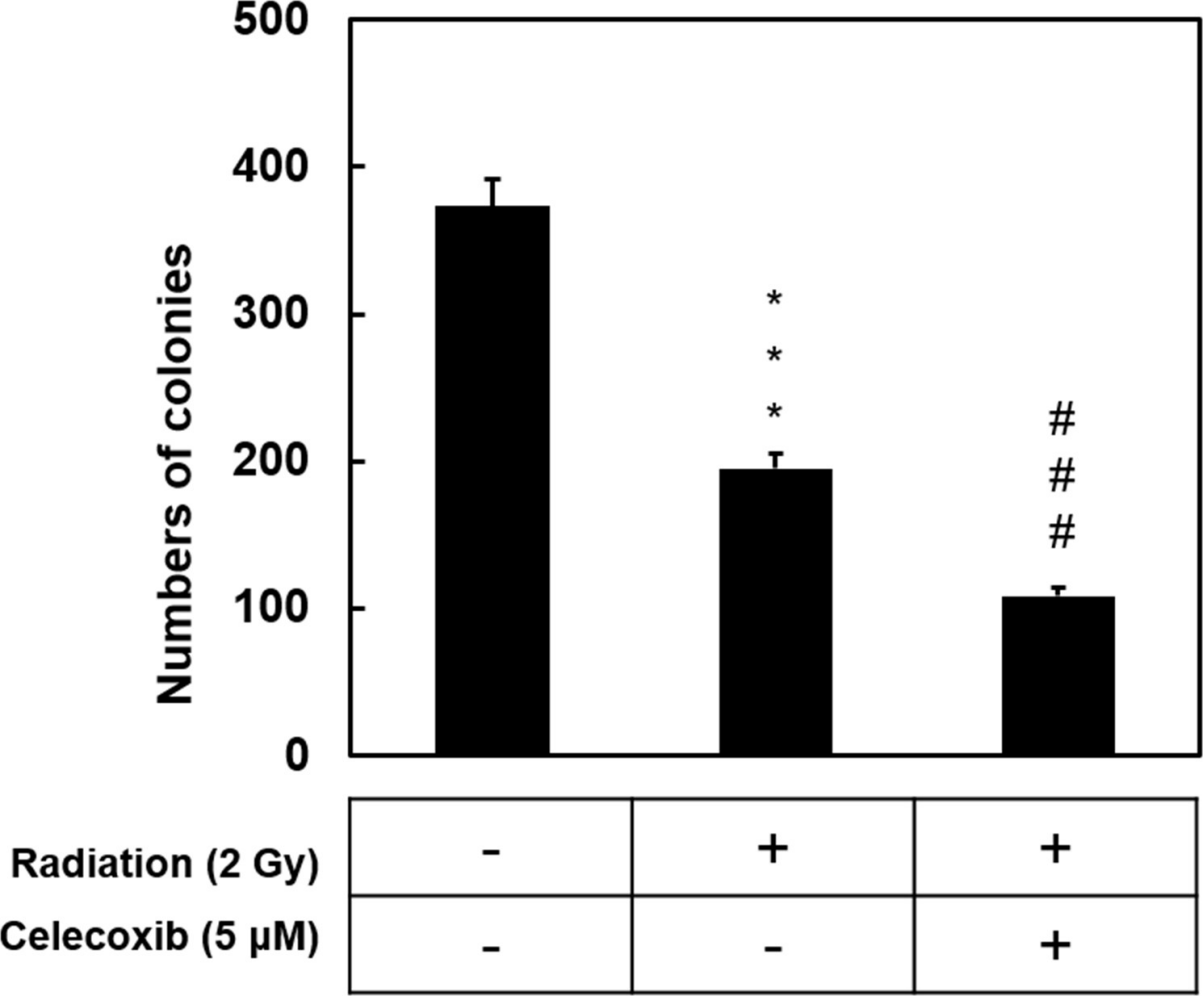
Synergistic effects of celecoxib and radiation on the inhibition of colony formation. Colony numbers were determined by counted under a microscope. Data are presented as the mean ± standard deviation (n = 3). ***P<0.001 vs. control; ^###^P<0.001 vs. radiation only.

In the cell viability assays it was observed that radiation caused a dose-dependent reduction in cell survival in all cell lines (Fig 1B–1E). DMSO or celecoxib alone had no significant effect on cell viability. Treatment with celecoxib (5 μM) for 24 h prior to radiation resulted in a significant decrease in cell viability compared with the celecoxib untreated but radiated samples. These findings indicated that celecoxib may induce a synergic effect of enhancing radiation efficacy. A549 cells exhibited a moderate effect against radiation challenge and were chosen for subsequent experiments. In Fig 2, data indicated that celecoxib treatment significantly reduced colony numbers of irradiated A549 cells compared with the radiation only group. Collectively, these results suggested that celecoxib enhanced the radiation sensitivity of NSCLC cells and current finding are consistent with previous publications [19, 22, 23].

### Celecoxib enhances radiation-induced apoptosis

**Fig 3:**
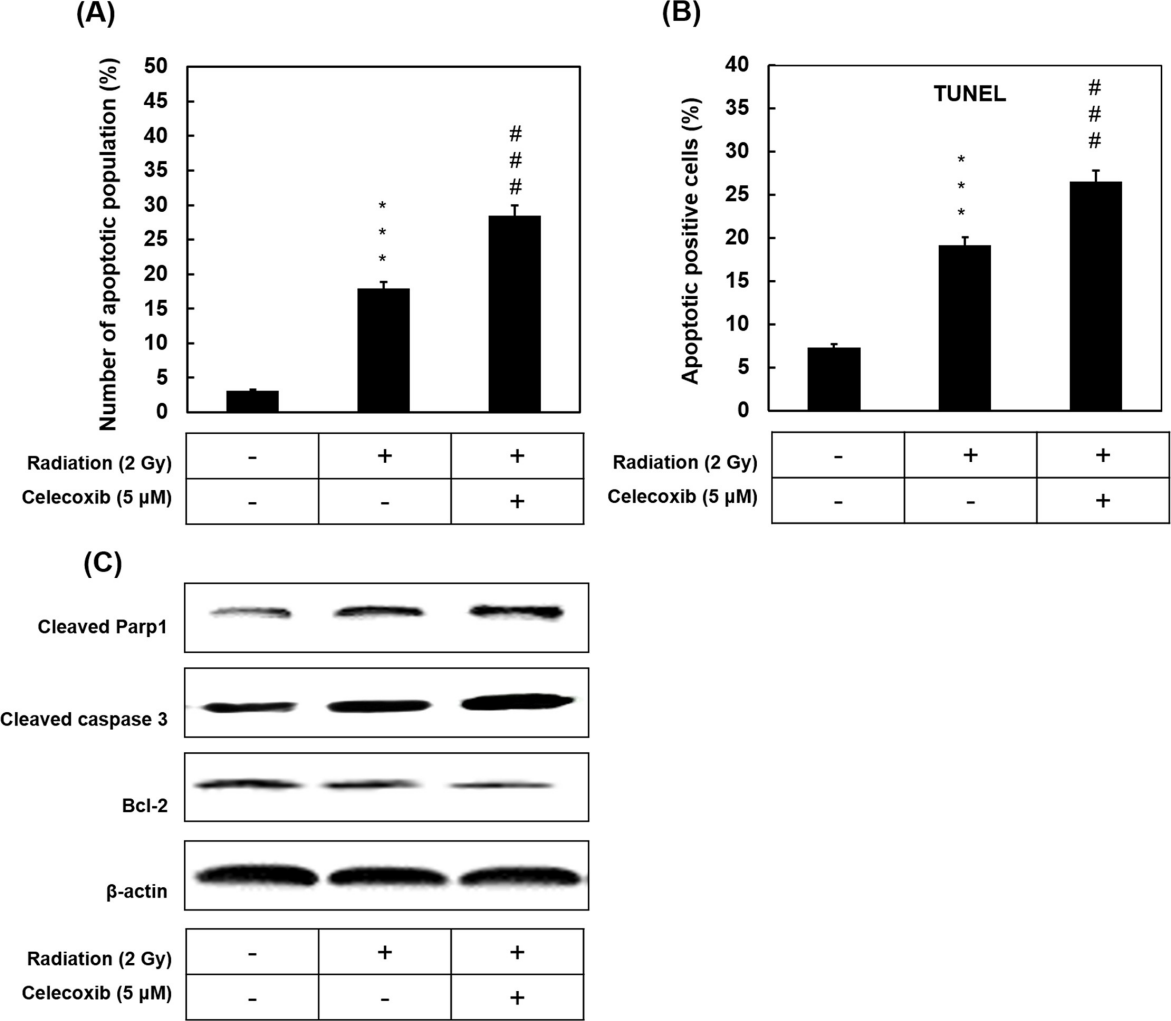
The effect of celecoxib and radiation on apoptosis. A549 cells were exposed to combination of Celecoxib and radiation for 24 h. (**A**) Cells were stained for Annexin V and PI staining and analyzed using flow cytometry, and the number of apoptotic population was presented. (**B**) Cells were fixed and analyzed for apoptotic cell death by TUNEL assay. Data were shown as the mean ± SD of three different experiments. ***P<0.001 indicated significant difference between control and radiation alone group, ^###^P<0.001 indicated significant difference between radiation alone with radiation plus Celecoxib groups. (**C**) Western blotting analysis of apoptosis-related protein expressions. β-Actin was used as loading control.

To investigate the induction of apoptosis following radiation, with or without celecoxib treatment, early apoptosis was determined by Annexin V/PI double staining. The percentage of early apoptotic cells significantly increased following radiation (2 Gy) compared with the untreated control (Fig 3A). Celecoxib treatment of radiated cells further significantly increased the radiation-induced apoptosis levels. Apoptotic effects of celecoxib and radiation in combination were evaluated using TUNEL assays. In accordance with the flow cytometry results, radiation significantly induced cell death compared with the untreated control and celecoxib treatment of radiated cells further significantly increased the numbers compared with the radiation group (Fig 3B). These results were confirmed by an assessment of apoptosis-related proteins. Western blotting result showed Parp1 cleavage, caspases 3 activation on treatment with Celecoxib in combination with radiation, in comparison to that observed in the groups treated with radiation alone. On the contrary, the expressions of anti-apoptotic Bcl-2 protein were clearly decreased in both groups and Celecoxib plus radiation group showed a greater extent than radiation treatment alone (Fig 3C).

### Celecoxib has no effect on DNA damage and cell cycle distribution in radiated NSCLC cells

**Fig 4:**
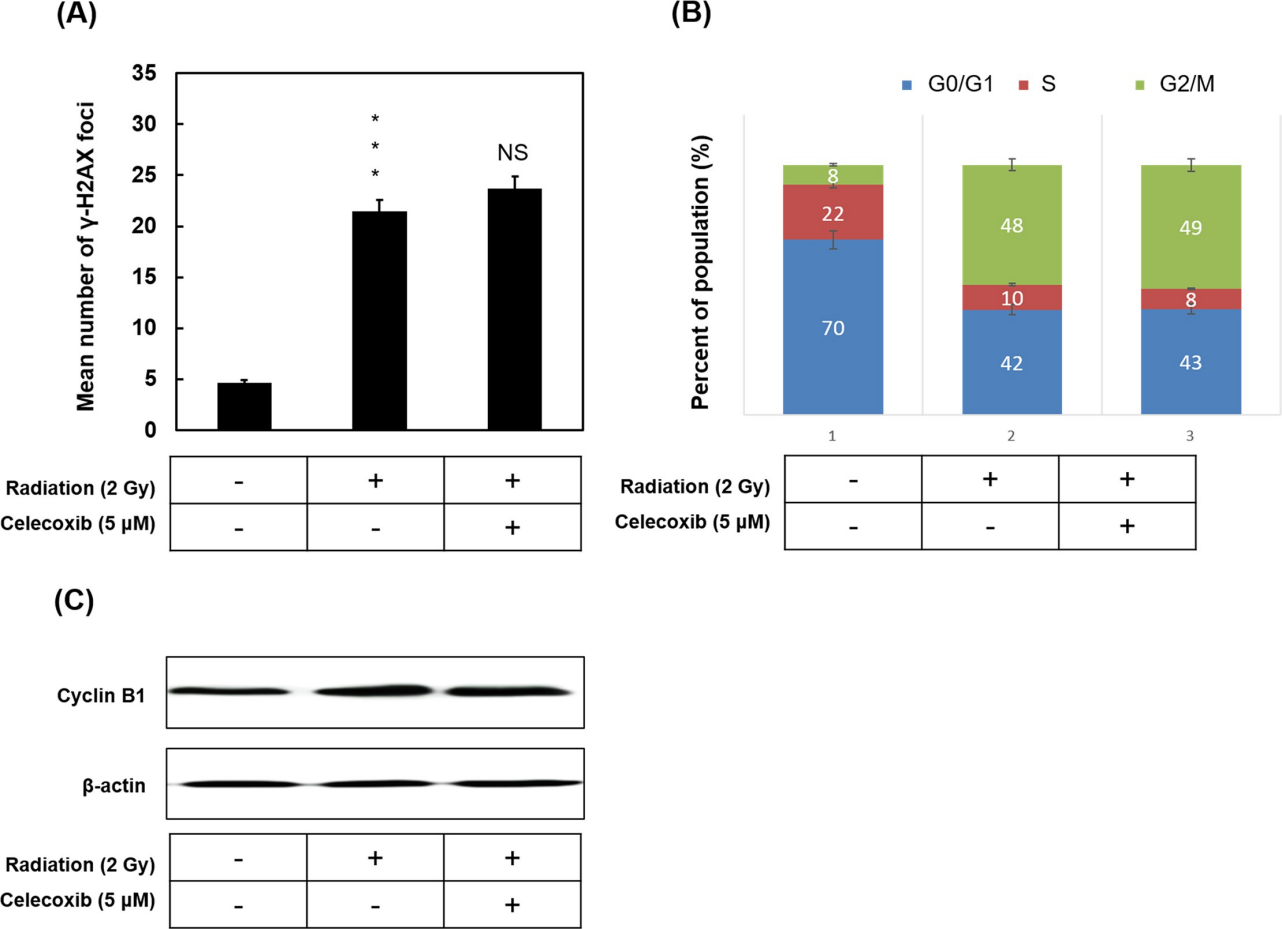
The effect of celecoxib and radiation on the formation of γ-H2AX foci and cell cycle distribution. (**A**) A549 cells were treated with or without 5 μM Celecoxib followed by radiation of 2 Gy for 24 h. Cells were fixed and labeled with anti-γ-H2AX primary antibody and Alexa Fluor 555 conjugated secondary antibody. γ-H2AX foci were observed by fluorescence microscopy. Nuclei were counterstained with DAPI. The number of γ-H2AX foci per cell was counted at least 50 cells for each condition. The average number was expressed as the mean ± SD of three different experiments. ***P<0.001 indicated significant difference between control and radiation alone group. NS, no significance. (**B**) Cell cycle distribution in A549 cells with radiation treatment in combination with or without Celecoxib, and then analyzed by the flow cytometer. (**C**) Western blotting analysis of Cyclin B1 protein. β-Actin was used as loading control. Data are presented as the mean ± standard deviation, n = 3. ***P<0.001 vs. control. γ-H2AX, phosphorylated histone H2AX.

To investigate what cellular mechanisms may enhance radiation-induced cell death following celecoxib treatment of radiated cells, effects of celecoxib on DNA damage and cell cycle populations were examined. As presented in Fig 4A, the number of γ-H2AX-positive foci was significantly enhanced by radiation compared with the untreated control. The combination of celecoxib with radiation resulted in a minimal increase and no significance was observed compared with the radiation only group. Other data indicated that radiation significantly altered the phase distribution of A549 cells compared with the untreated control (Fig 4B), with redistribution from the G0/G1 to the G2/M phases. The combination of celecoxib and radiation exhibited no significant changes compared with the radiation only group. Expression of cell cycle regulator cyclin B1 was assessed by western blot. As presented in Fig 4C, no significant changes in cyclin B1 expression were observed between the untreated, the radiation only and the combination groups.

### Celecoxib reverses radiation-induced effects on the AKT/mammalian target of rapamycin (mTOR) signaling pathway and COX-2 protein expression

**Fig 5:**
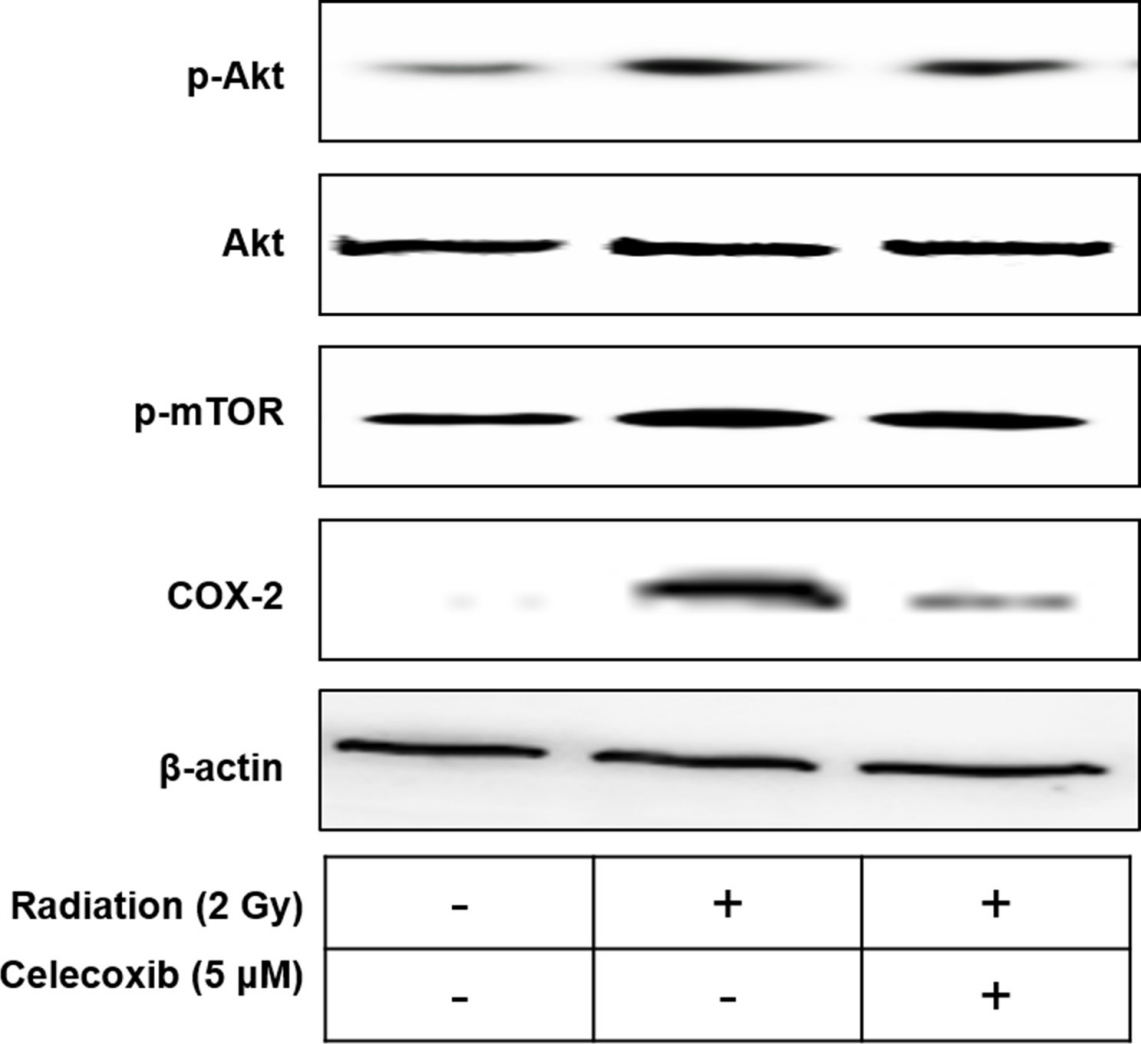
The effect of celecoxib and radiation on p-Akt, Akt, p-mTOR and COX-2 expressions in A549 cell lines. β-Actin was used as loading control.

As demonstrated in Fig 5, celecoxib treatment reduced radiation-induced phosphorylation of Akt and mTOR compared with the radiation only group. Celecoxib further attenuated COX-2 expression, which was elevated following radiation. These findings indicated that the mechanism of the radiosensitizing effect of celecoxib in A549 cells may be associated with the inhibition of radiation-induced activation of the AKT/mTOR signaling pathway and COX-2 expression.

### Celecoxib enhances tumor growth inhibition in a A549 radiated mouse model

**Fig 6:**
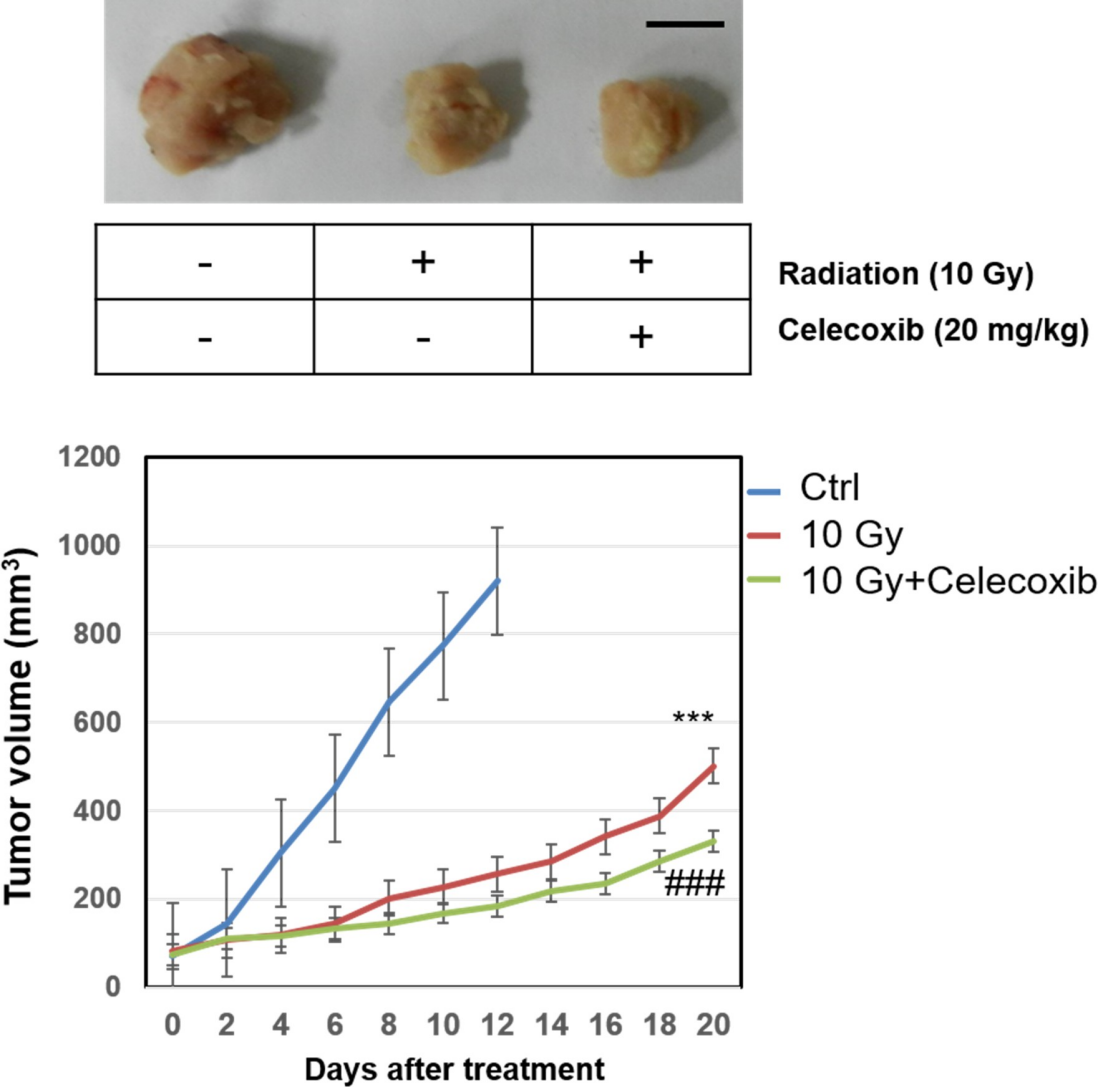
In vivo evaluation of the synergic effects of celecoxib and radiation therapy in A549 xenografts. Growth curves of lung cancer xenografts in nude mice established by injection of 1 × 10^7^ A549 cells in the flanks of nude mice and tumors were allowed to develop. Celecoxib was injected into the tail vein at day 0 when the mean tumor diameter was ~5 mm, and the tumor diameter was measured every other day. Irradiated at 10 Gy for three times with a 7-days of interval. Each group consisted of 6 animals and data are represented as mean ±SD. ***P<0.001 vs. control at day 10; ^###^P<0.001 vs. radiation only at day 20. Representative images of tumor in each group were shown (bar = 1 cm), tumor from control group was obtained from day 10, tumors from two other groups were from day 20.

**Fig 7:**
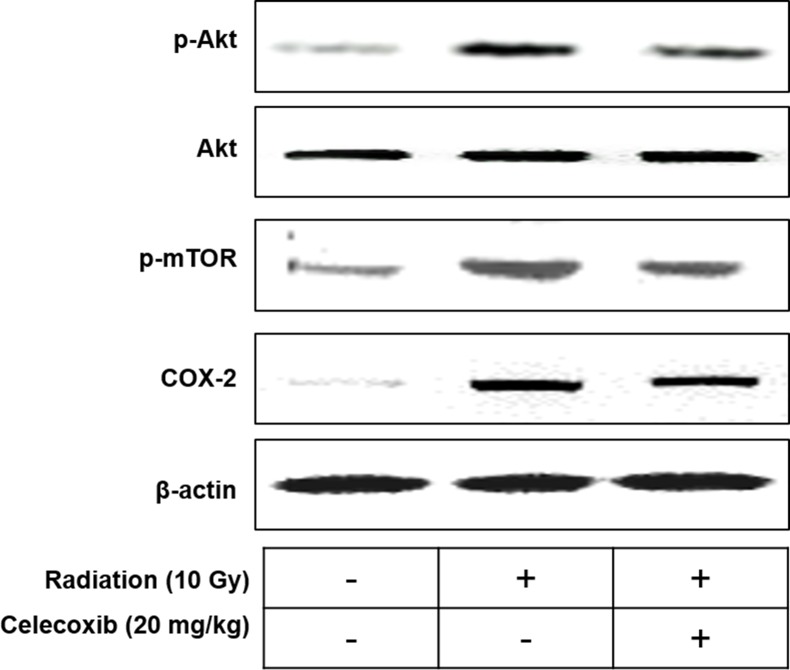
The effect of celecoxib and radiation on p-Akt, Akt, p-mTOR and COX-2 expressions in A549 xenograft animal models. β-Actin was used as loading control.

To confirm the radiosensitizing effect of celecoxib *in vivo*, a mouse model was established. Tumor volumes were significantly decreased in the radiosensitized mice compared with the untreated control at the endpoint of experiment (at day 20) (Fig 6). On day 10, the tumor volume was ~3-times higher in control mice compared with mice that received irradiation. The combination of radiation and celecoxib treatment decreased tumor volumes compared with the radiation only group (decreased by 22 and 35% on days 10 and 20, respectively). The tumor diameter for a single subcutaneous tumor was 1.75, 1.40 and 1.05 cm for the control, radiation only and combination treatment groups, respectively. We further investigated the Akt/mTOR signaling and COX-2 expression in tumors, as shown, similar results were obtained compare with in vitro experiments (Fig 7). The results suggested synergistic effects of the combination treatment.

## Discussion

Increasing the radiotherapy dose alone is not sufficient to increase the overall survival rate of cancerous patients. Therefore, there is an urgency to develop novel strategies to benefit the patients. For instance, the combination of radiosensitizers is a promising route. In the current study, it was revealed that NSCLC cell lines were affected by the co-treatment of celecoxib and radiation.

COX-2 inhibitors, including celecoxib, induce apoptosis at higher concentrations in NSCLC cell lines [24, 25] suggesting potential clinical applications for the combination of celecoxib with radio- and chemotherapy in NSCLC treatment [26]. However, trials reported contradictory results. A recent meta-analysis indicated that celecoxib induces adverse effects based on its therapeutic regimens or dosage [27]. The COX-2 inhibitor neither exaggerated nor antagonized the proapoptotic activity of chemotherapeutic agents in NSCLC cells [14, 28]. In addition, while certain reports support beneficial effects of combination treatment of celecoxib with radio- and chemotherapy, severe hematological toxicity cannot be omitted. A majority of clinical trials combined COX-2 inhibitors with chemo- rather than radiotherapy.

The role of celecoxib in cancer treatment has been extensively reviewed. The proapoptotic effect of celecoxib in friend's leukemia cancer cells has been attributed to COX-2 inhibition and subsequently restricts prostaglandin-mediated antiapoptotic protein expression [29]. Celecoxib regulates cell survival- and cell death-associated genes, including PDK-1, Akt, survivin, B-cell lymphoma 2 and myeloid leukemia cell differentiation protein 1 [30, 31]. Additionally to the role of celecoxib in COX-2 inhibition, mechanisms of proapoptotic effects of celecoxib have been reported. Individual studies have suggested that extrinsic and intrinsic apoptotic pathways are induced by celecoxib and associated compounds [32, 33]. The activation of the endoplasmic reticulum stress response is an early event for celecoxib-induced apoptosis [34].

Irradiation and antineoplastic drugs damage DNA; however, celecoxib-induced apoptosis is different and a putative benefit for the combination of celecoxib with radio- or chemotherapy has been suggested. Due to its selective interaction with COX-2, celecoxib is expected to pose less risk for gastrointestinal side effects and toxic effects of celecoxib have been attributed to the inhibitory action on COX-2. Therefore, the development of celecoxib derivatives without COX-2-inhibitory action describe a promising strategy for the treatment of COX-2-negative tumors [35]. It remains to be defined whether celecoxib and its derivatives may target apoptosis-associated genes.

In summary, the present study demonstrated that celecoxib exerted a radiosensitizing effect in radiated NSCLC cells by regulating the apoptosis-associated Akt/mTOR signaling pathway. The dosage of celecoxib used in the *in vivo* experiments combined with radiotherapy was decreased compared with current clinical standards and may have potential beneficial implications for patients with lung cancer.

## Abbreviations

COX-2: cyclooxygenase-2
γ-H2AX: phosphorylated histone H2AX
mTOR: mammalian target of rapamycin
Akt: protein kinase B
NSAID: nonsteroidal anti-inflammatory drug
NSCLC: non-small-cell lung cancer
TUNEL: terminal deoxynucleotidyl transferase dUTP nick-end labeling
