# Correction: Celecoxib enhances the sensitivity of non-small-cell lung cancer cells to radiation-induced apoptosis through downregulation of the Akt/mTOR signaling pathway and COX-2 expression

**DOI:** 10.1371/journal.pone.0224843

**Published:** 2019-10-30

**Authors:** The PLOS ONE Staff

There is an error in affiliation 2 for author Dan He. The correct affiliation 2 is: Department of Oncology, China National Nuclear Corporation 416 Hospital, Chengdu, Sichuan, P. R. China. The publisher apologizes for this error.
